# Case Report: Short-course defibrotide combined with eculizumab for TA-TMA

**DOI:** 10.3389/fimmu.2026.1771562

**Published:** 2026-04-24

**Authors:** Yan Yan, Xiaoning Li, Linyu Li, Shaoli Zhang, Liangming Ma, Lifang Huang, Weiwei Tian, Jie Zhao

**Affiliations:** 1 Shanxi Bethune Hospital, Shanxi Academy of Medical Sciences, Third Hospital of Shanxi Medical University, Tongji Shanxi Hospital, Taiyuan, China; 2 Tongji Hospital, Tongji Medical College, Huazhong University of Science and Technology, Wuhan, China

**Keywords:** defibrotide, eculizumab, HSCT, short-course, TA-TMA

## Abstract

Transplantation-associated thrombotic microangiopathy (TA-TMA) is a severe complication following hematopoietic stem cell transplantation, with its pathogenesis not yet fully understood and a lack of highly effective treatment options currently available. This article reports a case of a female patient with acute myeloid leukemia (AML) who was diagnosed with TA-TMA three months after a second allogeneic hematopoietic stem cell transplantation (allo-HSCT). After promptly discontinuing cyclosporine (CSA), and following ineffective treatments with anti-infective therapy, glucocorticoids, and plasma exchange, a short-course combination regimen—defibrotide (10 days) and eculizumab (3 weeks)—was administered. The TA-TMA-related indicators rapidly improved and returned to normal. During follow-up, the patient’s primary disease remained in remission, and she remained free of TA-TMA recurrence for five months. At eight months after the second allo-HSCT, TA-TMA recurred due to concurrent pneumonia and sepsis. The same short-course combination regimen—defibrotide (18 days) and eculizumab (3 weeks)—was administered, and the patient showed improvement in clinical and laboratory parameters again. This study is the first to report the use of a short-course combination therapy with defibrotide and eculizumab for TA-TMA. The combination regimen demonstrated good efficacy and tolerability, offering a potential treatment option for refractory TA-TMA, though its broader applicability requires further research and validation.

## Introduction

Acute myeloid leukemia (AML) is a highly heterogeneous group of hematopoietic malignancies characterized by abnormal clonal proliferation of myeloid hematopoietic stem cells, impaired differentiation, and resistance to apoptosis. Hematopoietic stem cell transplantation (HSCT) is a critical therapeutic approach for AML, particularly in intermediate-to-high-risk or relapsed/refractory cases. Transplantation-associated thrombotic microangiopathy (TA-TMA) is a severe complication following HSCT. Its primary clinical features include microangiopathic hemolytic anemia, thrombocytopenia, widespread microthrombosis, and multi-organ dysfunction (1). The pathogenesis of TA-TMA is complex and not yet fully elucidated, with current consensus suggesting a close association with endothelial cell injury after transplantation (2). Currently, the diagnosis of TA-TMA follows the unified criteria published by Schoettler et al (3). Regarding treatment, common strategies include supportive care, removal or adjustment of potential triggers (such as switching immunosuppressants), plasma exchange, defibrotide (which has endothelial protective effects), and eculizumab (targeting the complement pathway), although overall efficacy remains to be improved (1). Without timely intervention, the mortality rate of TA-TMA can be as high as 50–90% (4). Therefore, early recognition and aggressive treatment are crucial for improving patient prognosis.

This article reports the case of a 39-year-old female patient with AML who developed anemia, thrombocytopenia, elevated lactate dehydrogenase (LDH), schistocytes on peripheral blood smear, hypertension, elevated soluble C5b-9 levels (sC5b-9), and proteinuria within three months after the second allo-HSCT, leading to a diagnosis of TA-TMA. Upon diagnosis, cyclosporine (CSA) was immediately discontinued and switched to mycophenolate mofetil (MMF) for the prevention of graft-versus-host disease (GVHD). After poor responses to anti-infective therapy, glucocorticoids, and plasma exchange, a short-course therapy combining defibrotide (200 mg, every 12 hours, for 10 days) and eculizumab (900 mg/week, for 3 weeks) was initiated. The TA-TMA-related indicators rapidly improved and gradually returned to normal. Follow-up showed sustained remission of the primary disease, with no recurrence of TA-TMA for up to five months. Subsequently, the patient experienced a recurrence of TA-TMA due to concurrent pneumonia and sepsis. She was treated again with defibrotide (200 mg, every 8 hours, for 18 days) combined with eculizumab (900 mg/week, for 3 weeks), achieving another remission of TA-TMA. No significant drug-related adverse effects were observed during either treatment course. As of now, more than nine months have passed since the second allo-HSCT, and the patient’s primary disease remains in complete remission (CR).

## Case description

### Chief complaint and admission

A 39-year-old female patient was admitted in March 2025 due to “fever for 3 days, occurring more than 3 months after a second allo-HSCT for AML”.

Medical History:

In May 2023, the patient presented with fever and fatigue. A bone marrow aspiration biopsy, immunophenotyping, fusion gene analysis, and myeloid tumor gene mutation testing confirmed a diagnosis of AML with adverse prognosis (FLT3-ITD and KMT2A mutations, t (6;9) (p23;q34) DEK*::*NUP214). Complete remission was achieved after IA (IDA+Ara-C) induction chemotherapy, followed by two cycles of venetoclax plus intermediate-dose cytarabine chemotherapy. In November 2023, the patient underwent the first allo-HSCT (from a sibling haploidentical donor, compatible blood type, DSA negative). Continuous complete remission (CR) was maintained post-transplantation.

However, minimal residual disease turned positive in March 2024 (3.5 months post-transplant). The patient received one cycle of treatment with venetoclax, azacitidine, chidamide, and sorafenib, followed by oral sorafenib for maintenance therapy. In September 2024, the disease relapsed. Treatment with the Ara-C+HHT+G-CSF (CHG) regimen and CD123 CAR-NK cell therapy failed to induce remission. Subsequently, the FLT3 inhibitor gilteritinib was administered, achieving a CR with incomplete hematologic recovery (CRi).

In January 2025, a second allo-HSCT was performed (child to mother, HLA haploidentical). The conditioning regimen consisted of total body radiotherapy (TBI), busulfan (BU), fludarabine (Flu), and antithymocyte globulin (ATG). GVHD prophylaxis included CSA, MMF, and low-dose methotrexate (MTX). Neutrophil engraftment occurred on day +15, and platelet engraftment on day +28. Follow-up evaluation showed MRD negativity, complete donor chimerism by STR analysis, DEK*::*NUP214 0%, and FLT3-ITD allele ratio 0%. Gilteritinib maintenance therapy (80 mg/day) was initiated after hematologic stabilization at +2 months post-transplant.

### Current admission

On March 19, 2025 (day +48 post-allo-HSCT), the patient developed recurrent fever accompanied by dysuria and decreased urine output. Chest CT revealed multiple inflammatory infiltrates in both lungs. Urine culture identified carbapenem-resistant Klebsiella pneumoniae. Treatment with polymyxin B combined with ceftazidime-avibactam was initiated. The patient received a combination regimen including CSA (for GVHD prophylaxis), letermovir (for anti-cytomegalovirus), posaconazole enteric-coated tablets (for antifungal therapy). During this period, symptoms of limb numbness, dull headache, and elevated blood pressure (155–160/90–100 mmHg) emerged.

Laboratory findings showed pancytopenia (WBC 1.0×10^9^/L, Hb 65 g/L, PLT 14×10^9^/L). Peripheral blood smear revealed schistocytes. LDH was elevated (342.5 IU/L), with proteinuria (3.08 g/24h), an elevated urine microalbumin-to-creatinine ratio (623.33 mg/gCr), and increased soluble C5b-9 levels (357.17 ng/mL). Diagnosed as transplant-associated thrombotic microangiopathy (TA-TMA, BATAP score 4, high-risk) (5).

### TA-TMA treatment course

CSA was immediately discontinued and replaced with MMF combined with methylprednisolone (40 mg/day). Five sessions of plasma exchange (2000 ml each) were performed. Due to persistently high blood pressure (200/86 mmHg) and insufficient improvement in other symptoms and laboratory indicators, defibrotide (200 mg q12h for 10 days) combined with eculizumab (900 mg/week for a total of 3 doses) was added on April 7, 2025 (day +67 post-allo-HSCT).

**Figure 1:**
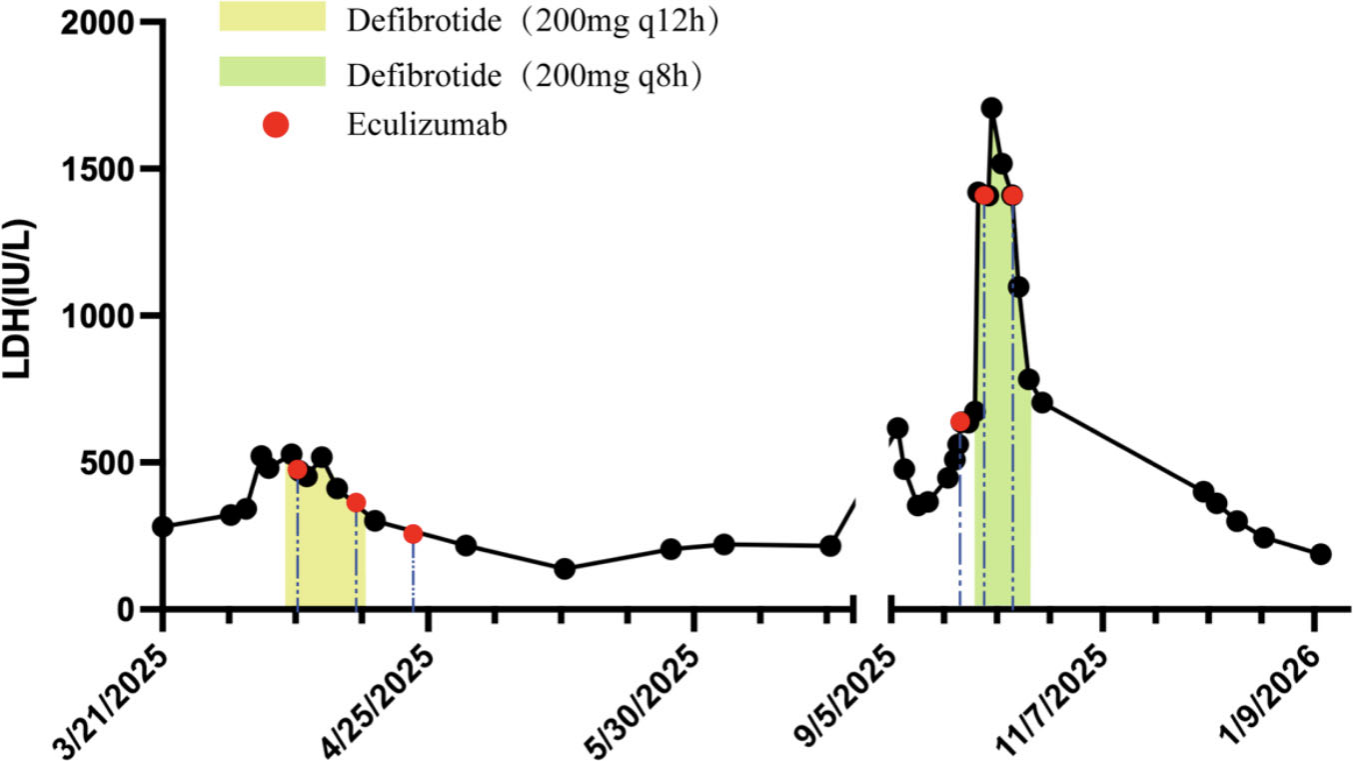
Serum lactate dehydrogenase (LDH) levels during the patient’s two hospital admissions (reference range: 120–250 IU/L). The yellow and green shaded areas represent the periods of defibrotide administration. Red dots indicate the timing of eculizumab doses.

**Figure 2:**
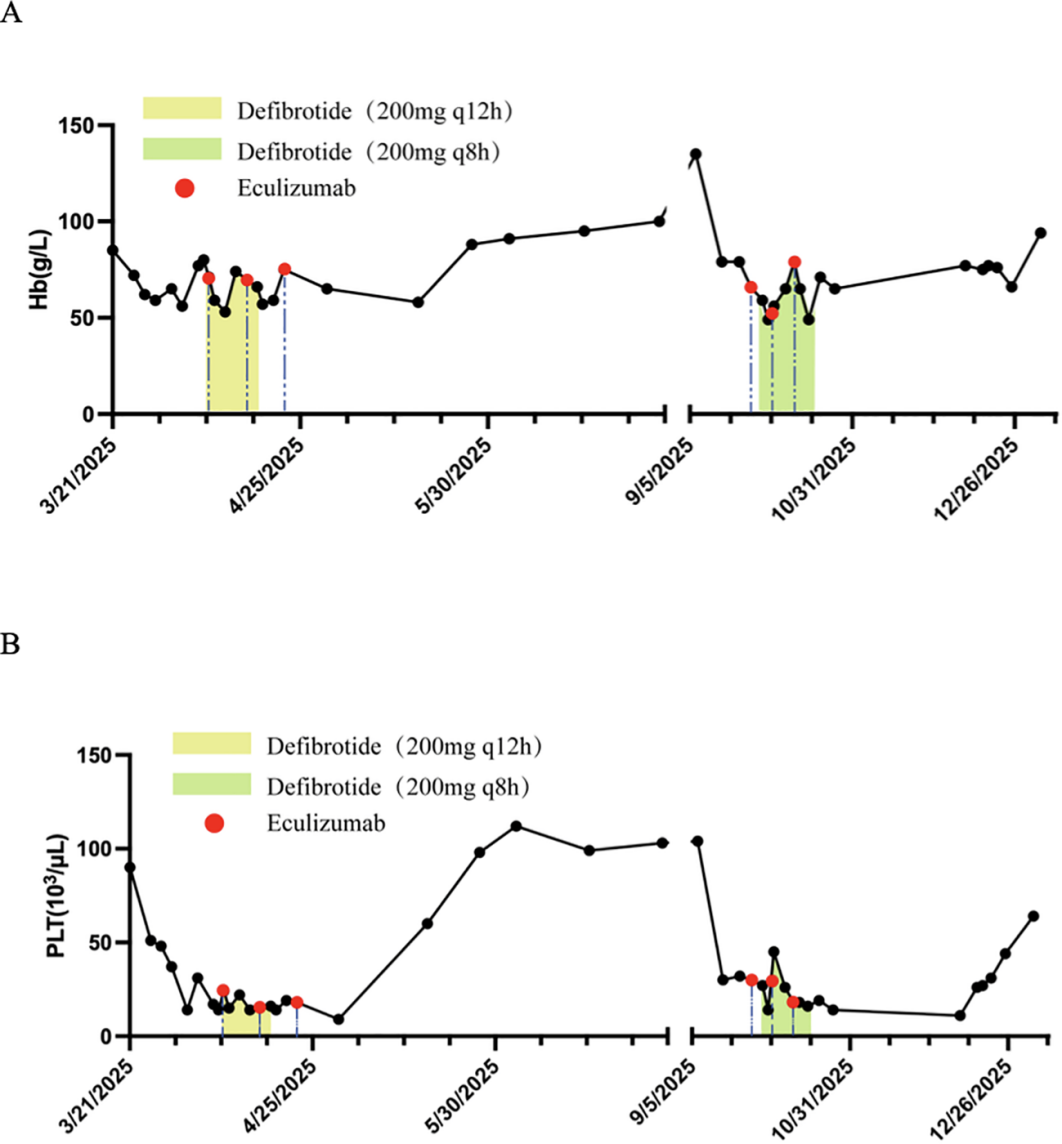
Changes in hemoglobin **(A)** and platelet **(B)** levels during the patient’s two hospital admissions. Reference ranges: hemoglobin (Hb) 115–150 g/L, platelets (PLT) 125-350×10³/μL. The yellow and green shaded areas represent periods of defibrotide administration. Red dots indicate the timing of eculizumab doses.

After ten days of combination therapy, blood pressure was controlled. Laboratory parameters including LDH, sC5b-9, and urinary protein returned to normal on days +30, +35, and +39, respectively (LDH: 217 IU/L, see **Figure 1**; sC5b-9: 195.01 ng/ml; urinary protein: 0.45 g/24h). Blood counts recovered and stabilized after 47 days (see **Figure 2**). Re-evaluation of the primary disease showed sustained CR in the bone marrow, with MRD negativity and negative FLT3 status.

The patient continued maintenance therapy with gilteritinib 80 mg daily after discharge. In August 2025 (day +213 post-allo-HSCT), follow-up testing showed normal levels of the complement complex sC5b-9 (113.65 ng/mL). The patient received vaccinations against meningococcus and Streptococcus pneumoniae while under outpatient care.

### Recurrence and retreatment of TA-TMA

In September 2025 (day +220 post-allo-HSCT), the patient presented with fever and chest pain. Bronchoalveolar lavage fluid next-generation sequencing (BALF-NGS) identified cytomegalovirus pneumonia and Pneumocystis jirovecii pneumonia. During this period, the patient developed Escherichia coli sepsis. Based on the above findings, anti-infective therapy was administered, including ganciclovir for cytomegalovirus, caspofungin for Pneumocystis jirovecii, and a combination of ceftazidime-avibactam and aztreonam to cover bacterial infection. During this period, pancytopenia was observed (WBC 1.3×10^9^/L, neutrophils 0.46×10^9^/L, Hb 64 g/L, platelets 32×10^9^/L). Therefore, ganciclovir was discontinued and replaced with foscarnet sodium for continued anti-cytomegalovirus therapy. In addition, trimethoprim-sulfamethoxazole was combined to enhance coverage against Pneumocystis jirovecii pneumonia. Thereafter, the patient developed elevated blood pressure (145–160/70–100 mmHg), accompanied by dizziness, and headache. Schistocytes were noted on peripheral blood smear, with LDH elevated to 1707 IU/L (see **Figure 1**), sC5b-9 level at 266.21 ng/mL, and urine microalbumin-to-creatinine ratio increased to 373.9 mg/gCr, indicating recurrence of TA-TMA.

Retreatment with defibrotide (200 mg q8h for 18 days) combined with eculizumab (900 mg/week for a total of 3 doses) was initiated. Following treatment, symptoms resolved, and normalization of sC5b-9 (118.91 ng/mL) and LDH (244 IU/L) levels was observed on days +18 and +108 after treatment initiation, respectively (see **Figure 1**). Blood pressure was controlled, and blood counts (hemoglobin and platelets) gradually recovered on day +92 after treatment initiation (see **Figure 2**).

### Follow-up

As of January 2026, 12 months after the second transplant, the patient remains in sustained CR for AML, with negative FLT3-ITD mutation, negative DEK*::*NUP214 fusion gene, and complete donor chimerism by STR analysis. No recurrence of TA-TMA has been observed. During the treatment period, no significant drug-related adverse reactions such as severe organ bleeding, central nervous system infection, thrombosis, or organ dysfunction were noted.

## Discussion

TA-TMA is one of the most challenging complications following hematopoietic stem cell transplantation. Its pathological mechanism is complex, involving a vicious cycle of endothelial injury, abnormal activation of the complement system, inflammatory responses, and microthrombosis (1). Despite increasing understanding in recent years, clinical management remains a significant challenge, particularly for refractory cases that do not respond to conventional treatments. This study is the first to report the successful treatment of a high-risk TA-TMA patient using a short-course, low-dose combination regimen of defibrotide and eculizumab, with efficacy reconfirmed during an infection-induced recurrence. This successful case suggests that this combination strategy may represent a promising new option for treating refractory TA-TMA.

A systematic differential diagnosis was performed at the time of both TA-TMA episodes:

Although ADAMTS13 activity was not measured, the likelihood of thrombotic thrombocytopenic purpura (TTP) was very low. The presence of proteinuria, persistent hypertension, and elevated sC5b-9 levels did not support TTP but instead suggested complement-mediated TA-TMA. The favorable response to eculizumab further supported this diagnosis.

Although genetic testing was not performed and hereditary complement abnormalities cannot be fully excluded, the absence of prior TMA episodes or family history, together with clear transplant-related triggers, favors secondary complement activation rather than primary atypical hemolytic uremic syndrome (aHUS).

While GVHD may coexist, the presence of severe microangiopathic hemolytic anemia, thrombocytopenia, renal involvement, and elevated sC5b-9 indicates that TA-TMA was the predominant process. Taken together, based on the clinical context, laboratory findings, and treatment response, the diagnosis of TA-TMA was established.

Furthermore, the patient received gilteritinib maintenance therapy post-transplantation. However, to date, no clinical studies or case reports have directly confirmed that highly selective FLT3 inhibitors such as gilteritinib can induce or exacerbate TA-TMA. Unlike multi-kinase inhibitors like sorafenib and sunitinib, gilteritinib lacks significant cross-inhibitory effects on the VEGFR family and theoretically poses a lower risk of direct endothelial toxicity. Therefore, we consider gilteritinib an unlikely contributor to the development of TA-TMA in this case.

The therapeutic outcome in this case is encouraging. After discontinuing cyclosporine and receiving standard treatments such as anti-infective therapy and glucocorticoids, the patient’s TA-TMA indicators remained inadequately controlled. However, following the addition of the short-course, low-dose combination therapy with defibrotide and eculizumab, key biomarkers such as sC5b-9 normalized within a short period, and other laboratory parameters improved sequentially, with no significant adverse effects observed during either treatment course. These results indicate that this combination regimen may exert synergistic therapeutic effects, potentially leading to more profound and rapid pathophysiological reversal compared to monotherapy.

From a mechanistic perspective, this synergy arises from the precise intervention of the two drugs targeting different aspects of the TA-TMA pathological process. Eculizumab, as an anti-C5 monoclonal antibody, specifically blocks the terminal complement pathway, effectively inhibiting the formation of the membrane attack complex (MAC) and thereby controlling “downstream” hemolytic reactions and endothelial cell lysis (6). In contrast, defibrotide acts on more “upstream” pathological processes through its multifaceted pharmacological effects—including promoting fibrinolysis (upregulating tPA and downregulating PAI-1), antithrombotic activity, anti-inflammatory actions (inhibiting leukocyte-endothelial adhesion), antioxidant properties, and direct endothelial protection—thereby comprehensively stabilizing the vascular endothelial barrier (7). The combination of these two agents establishes a complete defense line, spanning from upstream protection to downstream inhibition, potentially more effectively interrupting the “vicious cycle” of TA-TMA.

Literature data indicate that monotherapy for TA-TMA has certain limitations. Jodele et al (6) reported a one-year response rate of 64% and an overall survival rate of 66% with eculizumab treatment in high-risk pediatric TA-TMA patients. A multicenter prospective study showed that nearly three-quarters (73%) of TA-TMA patients treated with eculizumab achieved comprehensive recovery of organ function and maintained good overall health. Meta-analysis results demonstrated that eculizumab treatment significantly improves both overall survival and clinical response rates in TA-TMA patients, with drug-related adverse events occurring at an acceptable level (8). In studies of defibrotide monotherapy for TA-TMA, retrospective data revealed a median time to response of 14 days and a complete response rate of 65% after defibrotide administration in TA-TMA patients (9). In this case, the combination therapy not only achieved rapid remission but also induced remission again following recurrence triggered by infection—a strong precipitating factor—suggesting that combination therapy may offer a new option for patients who respond inadequately to traditional monotherapy.

Particularly noteworthy is the unique clinical value demonstrated by the short-course strategy employed in this case. Compared to traditional long-term treatment regimens, the short-course combination therapy significantly reduced the risk of encapsulated bacterial infections (especially meningococcal infections) associated with prolonged complement suppression, an advantage particularly important for immunocompromised post-transplant patients. Furthermore, the shortened treatment duration directly reduced the total drug dosage and treatment length, substantially lowering medical costs and hospitalization burden, thereby improving treatment accessibility.

In this case, TA-TMA recurred five months after the initial remission due to severe pneumonia and sepsis, a clinical course that highlights the complex relationship between infection and TA-TMA. Existing research has shown that pathogen infections, such as cytomegalovirus, can directly cause endothelial cell injury by activating monocytes and T lymphocytes, promoting the release of pro-inflammatory cytokines like TNF-α and IL-1, thereby inducing or exacerbating TMA (10). The recurrence in this case further confirms infection as a significant precipitating factor for TA-TMA, while also reflecting that the combination therapy remained effective even in the presence of a strong trigger. Notably, during the second treatment, we appropriately intensified the defibrotide dosage (from q12h to q8h) and extended the treatment duration—an adjustment based on individualized assessment of the patient’s disease severity. The final efficacy confirmed the rationality of this strategy. Such flexible adjustments in dosage and duration provide a reference for further optimizing treatment regimens.

As a single case report, this study has inherent limitations. The follow-up period remains relatively short; whether this regimen can prevent TA-TMA recurrence triggered by non-infectious factors, as well as its long-term efficacy and safety, requires longer-term observation. Notably, TA-TMA exhibits significant heterogeneity in pathophysiology and severity. Different triggers (e.g., drug toxicity, infection, graft-versus-host disease) may lead to distinct mechanisms of endothelial injury and complement activation pathways. Furthermore, the patient’s genetic background, underlying diseases, and the stage of TA-TMA progression can also influence clinical manifestations and treatment responses. The successful case in this study may not be fully generalizable to all TA-TMA patients, particularly those with different underlying etiologies, more severe organ dysfunction, or poor responsiveness to complement inhibitors. Therefore, caution is warranted when extrapolating the results of this study to a broader population of TA-TMA patients, and individual differences should be fully considered. Given these limitations, prospective studies are warranted in the future to validate the applicability of this approach.

## Conclusion

This study is the first to report the successful application of a short-course regimen combining defibrotide and eculizumab in the treatment of refractory TA-TMA. Through multi-target synergistic effects, this regimen demonstrated rapid and significant therapeutic efficacy in two independent episodes, with good tolerability. Its short-course characteristic offers unique advantages in reducing infection risk, alleviating economic burden, and preserving immune function. Although, as a single-case experience, its broader application requires support from larger-scale studies, in the current context of limited treatment options for TA-TMA, this regimen provides a valuable therapeutic approach for clinically refractory cases. Patients who may benefit the most include those with early diagnosis, clear evidence of complement activation, concurrent bone marrow suppression, or high infection risk. Future research should focus on stratified analysis based on different pathophysiological mechanisms and severity levels, in order to clarify the applicable population, optimal initiation timing, and treatment duration of this short-course combination strategy. This will provide more reliable evidence for the individualized and precise treatment of TA-TMA.

## Data Availability

The original contributions presented in the study are included in the article/Supplementary Material. Further inquiries can be directed to the corresponding authors.
